# Distribution of *HLA-ABC* allele groups in a cohort of Chilean rheumatoid arthritis patients and healthy individuals

**DOI:** 10.1186/s40659-025-00663-w

**Published:** 2025-12-24

**Authors:** Miqueas Jaime, Lucero Toro, Constanza Varela-Villarroel, Darly Montano-Bruno, Bárbara Pesce, Daniela Schneider, Lilian Soto, Francisca Bozán, Óscar Neira, María C. Cuéllar-Gutiérrez, Consuelo Arroyo, Guido Rivera, Eduard Palou, Diego Catalán, Jaxaira Maggi, Juan C. Aguillón

**Affiliations:** 1 Immune Regulation and Tolerance Research Group (IRT Group), Núcleo Interdisciplinario de Farmacología e Inmunología, Instituto de Ciencias Biomédicas (ICBM), Facultad de Medicina, Universidad de Chile, Santiago, Chile; 2 MED.UCHILE-FACS Lab REDECA, Instituto de Ciencias Biomédicas (ICBM), Facultad de Medicina, Universidad de Chile, Santiago, Chile; 3 Hospital Clínico de la Universidad de Chile, Santiago, Chile; 4 Hospital del Salvador, Santiago, Chile; 5 Servicio de Inmunología, Hospital Clínic de Barcelona, Barcelona, Spain; 6 Biological and Environmental Proteomics Group, IIBB-CSIC, Barcelona, Spain

**Keywords:** Rheumatoid arthritis, *HLA-ABC* and *HLA-DRB1* alleles, Shared epitope

## Abstract

**Background:**

Rheumatoid Arthritis (RA) is an autoimmune disease in which *HLA-DRB1* alleles encoding the “Shared Epitope” (SE), located in the β-chain of class II HLA-DR molecules, constitute the main genetic risk factor. However, there is scarce information about the role of HLA class I genes (*HLA-ABC*) in RA susceptibility. The present work aimed to evaluate the distribution of *HLA-ABC* allele groups in a cohort of Chilean RA patients and healthy subjects (HS), and to explore the influence of *HLA-DRB1* SE alleles on this distribution.

**Results:**

135 RA patients and 122 HS were genotyped for *HLA-ABC*. The most frequent allele groups were HLA-A*02 (24.0%), HLA-B*39.1 (14.2%), and HLA-C*07 (24.7%) for RA patients, and HLA-A*02 (31.5%), HLA-A*24 (12.8%) and HLA-C*07 (17.7%) for HS. RA patients presented a significantly higher frequency of HLA-C*07 (*p* = 0.0015) and HLA-B*39.1 (*p* = 0.037) allele groups compared to HS. After applying the Bonferroni correction, the significant difference remained only for the HLA-C*07 allele group (*p* = 0.015). In a subset of RA patients (n = 60), positive for *HLA-DRB1* SE alleles, the most frequent *HLA-ABC* allele groups were HLA-A*02 (0–33.3%), HLA-B*39.1 (0–16.7%), and HLA-C*07 (22.2–60.0%), whereas HLA-B*39.2 and HLA-B*52 were the least frequent ones. Overall, HLA-C*07 was the most frequent allele group across RA patients carrying *HLA-DRB1* SE alleles.

**Conclusions:**

The HLA-C*07 allele group shows a significantly higher presence in RA patients compared to HS. In contrast, the distribution of most other *HLA-ABC* allele groups in this cohort displays a similar frequency between RA patients and HS, consistent with data from different populations.

**Supplementary Information:**

The online version contains supplementary material available at 10.1186/s40659-025-00663-w.

## Background

Rheumatoid Arthritis (RA) is a chronic inflammatory autoimmune disease of unknown etiology that primarily affects the joints, frequently evolving towards joint destruction and deformity over time [1]. The global prevalence of RA varies geographically, with higher rates reported in industrialized countries and an overall estimate of 0.46% between 1980 and 2019 [2], while in Chile the prevalence is 0.6% [3]. It has also been reported that RA is two to three times more frequent in women than in men, with an increasing incidence during the last decades, and that sex hormones are responsible in part for this difference [4, 5].

Although environmental factors, such as microbiota composition, have been shown to contribute to RA pathogenesis, genetic influences are currently better characterized [6]. Among the genetic factors, there is overwhelming evidence linking RA to genes of the classical major histocompatibility complex (MHC) [7], known in humans as Human Leukocyte Antigen (HLA), a 3.6 megabase-pair (Mb) region of the genome located on the short arm of chromosome 6 (6p21). This region of the human genome contains genetic *loci* that encode for class I (HLA-A, -B, and -C) and class II proteins (HLA-DP, -DQ, and -DR), among many other genes that have been discovered, leading to the definition of x-MHC, which corresponds to the extended version with a length of 7.6 Mb [8]. Thus, research conducted over several decades has demonstrated a pivotal contribution to disease pathogenesis of *HLA-DRB1* alleles encoding the “Shared Epitope” (SE) (*HLA-DRB1* SE), a conserved amino acid sequence located at positions 70–74 in the β-chain of the class II HLA-DR molecule [9, 10], specifically among RA patients positive for disease-specific anti-citrullinated protein antibodies (ACPAs) [11–13]. More than 70% of RA patients are ACPA-positive, a status that correlates with disease severity [14].

Despite minimal variations in the global prevalence of RA, important differences have been described for *HLA-DRB1* SE allele frequencies between different ethnic groups. Thus, the HLA-DRB1*04:01 and HLA-DRB1*04:04 alleles are frequent in Caucasian RA patients [6, 15, 16], whereas the HLA-DRB1*04:05 allele is more widely distributed among East Asian populations [17–20]. Of note, the non-SE HLA-DRB1*09:01 allele confers increased risk for ACPA-positive RA in Japanese and Korean populations [18, 21]. In Chilean RA cohorts, in addition to the already mentioned alleles, the HLA-DRB1*01:01, HLA-DRB1*10:01, and HLA-DRB1*04:08 alleles have been associated to an increased risk [22, 23].

In contrast, much less is known about the association of HLA-ABC molecules with RA, likely because RA has been described as primarily mediated by CD4+ T cells recognizing antigens presented by HLA-DR molecules [24]. Nonetheless, more recently, a single nucleotide polymorphism (SNP) involving a substitution of histidine or tyrosine for asparagine at position 9 in the alpha chain peptide-binding groove of class I HLA-B molecule (HLA-B*8-Asp9), corresponding to the HLA-B*08:01 allele, has been associated with a higher risk for ACPA-positive RA [17, 25].

Although it has already been shown that *HLA-ABC* and *HLA-DR* allele frequencies and haplotypes differ significantly across ethnicities, the most prevalent *HLA-ABC* allele groups remain relatively consistent. Thus, studies across European, Asian, and Latin American populations have reported that, for each locus, the predominant allele groups are HLA-A*02, HLA-A*24, and HLA-A*01; HLA-B*39, HLA-B*35, and HLA-B*40; and HLA-C*07, HLA-C*04 and HLA-C*01 [26–31]. A similar distribution has been described in the Chilean population, where allele groups HLA-A*02, HLA-B*39, and HLA-C*07 are the most frequent [32–34].

In this work, we examined whether the distribution of the most prevalent *HLA-ABC* allele groups across populations differs between cohorts of RA patients and healthy subjects (HS) from the Metropolitan Region of Santiago, Chile. Furthermore, we evaluated the distribution of these *HLA-ABC* allele groups in relation to the presence of *HLA-DRB1* SE alleles in RA patients.

## Methods

### Study participants

To conduct this cross-sectional study, blood samples from 135 RA patients and 122 HS were collected between 2014 and 2025. Peripheral blood samples were obtained by venipuncture from RA patients and HS from the Hospital Clínico Universidad de Chile and Hospital del Salvador after participants signed a written informed consent in accordance with the Declaration of Helsinki. All patients met the American College of Rheumatology (ACR) and European League Against Rheumatism (EULAR) classification criteria for diagnosing RA [35]. The average age of RA patients and HS was 58.1 and 37.7 years, while the female/male gender percentages were 87.4/12.6% and 57.4/42.6%, respectively. For comparisons of *HLA-ABC* and *HLA-DRB1* allele groups, a sub-cohort of 60 RA patients was defined, all of whom carried the SE, and of these, 90.2% had circulating ACPAs. All procedures were approved by the Ethics Committees of each involved institution.

### Genomic DNA extraction

Genomic DNA extraction was performed using the “Salting Out” procedure. Briefly, 1 mL of a Tris-based buffer (pH 7.6) containing HCl, KCl, MgCl_2_, and EDTA, was placed in a 15 mL conical tube. Next, 1% Triton X-100 was added, and the mixture was gently pipetted and briefly vortexed. One milliliter of whole blood was then added and centrifuged at 2,500xg for 10 min at room temperature. After discarding the supernatant, the pellet was resuspended in the same Tris-based buffer and centrifuged at 500xg for 10 min. The resulting pellet was resuspended in a second Tris-based buffer containing HCl, KCl, MgCl_2_, NaCl and EDTA. After adding 10% SDS and mixing by pipetting until a viscous consistency appeared, proteinase K (10 ng/μl) was added, gently mixed by inversion, and incubated at 55 °C for 10 min. Subsequently, 6 M NaCl was added, mixed thoroughly by inversion, and centrifuged at 3,200xg for 30 min. The supernatant was recovered and transferred to a new 15 mL tube and two volumes of 100% ethanol were added and mixed by inversion until a “white cloth” precipitate was formed. The content was then transferred to a microcentrifuge tube containing 1 mL of 70% ethanol and centrifuged at 10,000xg for 5 min. After removing the supernatant, 100 μl of a Tris–EDTA buffer (pH 8.0) was used to resuspend the pellet by pipetting. The DNA concentration was measured with a NanoDrop spectrophotometer and yields above 100 ng/μl with an A260/A280 ratio greater than 1.8 were considered optimal.

### HLA typing

*HLA-ABC* allele groups were determined by PCR using sequence-specific primers (PCR-SSP) targeting the HLA class I region, enabling the detection of specific allele groups, as listed in Supplementary Table 1. Briefly, 0.75 μl each of sequence-specific sense and anti-sense primers (both at 10 μM), along with an internal control pair of primers, were added to the PCR tubes. Next, 1 μl of DNA at 100 ng/μl was included, followed by GoTaq® G2 Master Mix (Promega). The amplification cycles were as follows: 1 cycle at 96 °C for 60 s; 5 cycles at 96 °C for 25 s, 70 °C for 45 s, and 72 °C for 25 s; 21 cycles at 96 °C for 25 s, 65 °C for 50 s, and 72 °C for 30 s; 4 cycles at 96 °C for 20 s, 55 °C for 60 s, and 72 °C for 90 s; and 1 cycle at 20 °C for 30 s. The PCR products were separated by electrophoresis. Samples were loaded on a 1.5% (w/v) agarose gel bed, along with the 1 Kb molecular weight standard (MaestroGen Inc.). Tris–acetate-EDTA buffer was used as a running buffer for the electrophoresis, which was carried out at 90 V for 45 min. Finally, the DNA bands were visualized in a UV transilluminator using the SafeView Plus probe (Fermelo Biotec, Chile).

*HLA-DRB1* alleles were determined by the PCR-SSO (Sequence Specific Oligonucleotides) method reverse technique, following the manufacturer’s instructions (Tepnel Lifecodes Corporation). Briefly, *HLA-DRB1* exon 2 was amplified and subsequently hybridized with DNA probes covering the main polymorphic positions of the gene, providing an approximation of the most probable alleles (though not offering definitive high-resolution confirmation). A Luminex instrument was used for data acquisition.

### Statistical analysis

The frequency of *HLA-ABC* allele groups in HS and RA patients was compared using the Chi-square test or Fisher’s exact test. Odds ratios (ORs) were calculated with 95% confidence intervals (CI) to study associations between the disease and *HLA-ABC* allele groups. *p*-values ≤ 0.01 and 0.05 were considered statistically significant. Those differences that were statistically significant were tested using the Bonferroni correction for multiple comparisons to control for Type I error when performing multiple statistical tests on the same data. Only those associations that remained below the adjusted threshold were considered significant. GraphPad Prism version 10.0.0 for Windows (GraphPad Software, Boston, Massachusetts USA, www.graphpad.com) and IBM SPSS Statistics version 29.0.2.0 were used for statistical analysis and graphing.

## Results

### Frequency of *HLA-ABC* allele groups in Chilean RA patients and HS

**Fig. 1:**
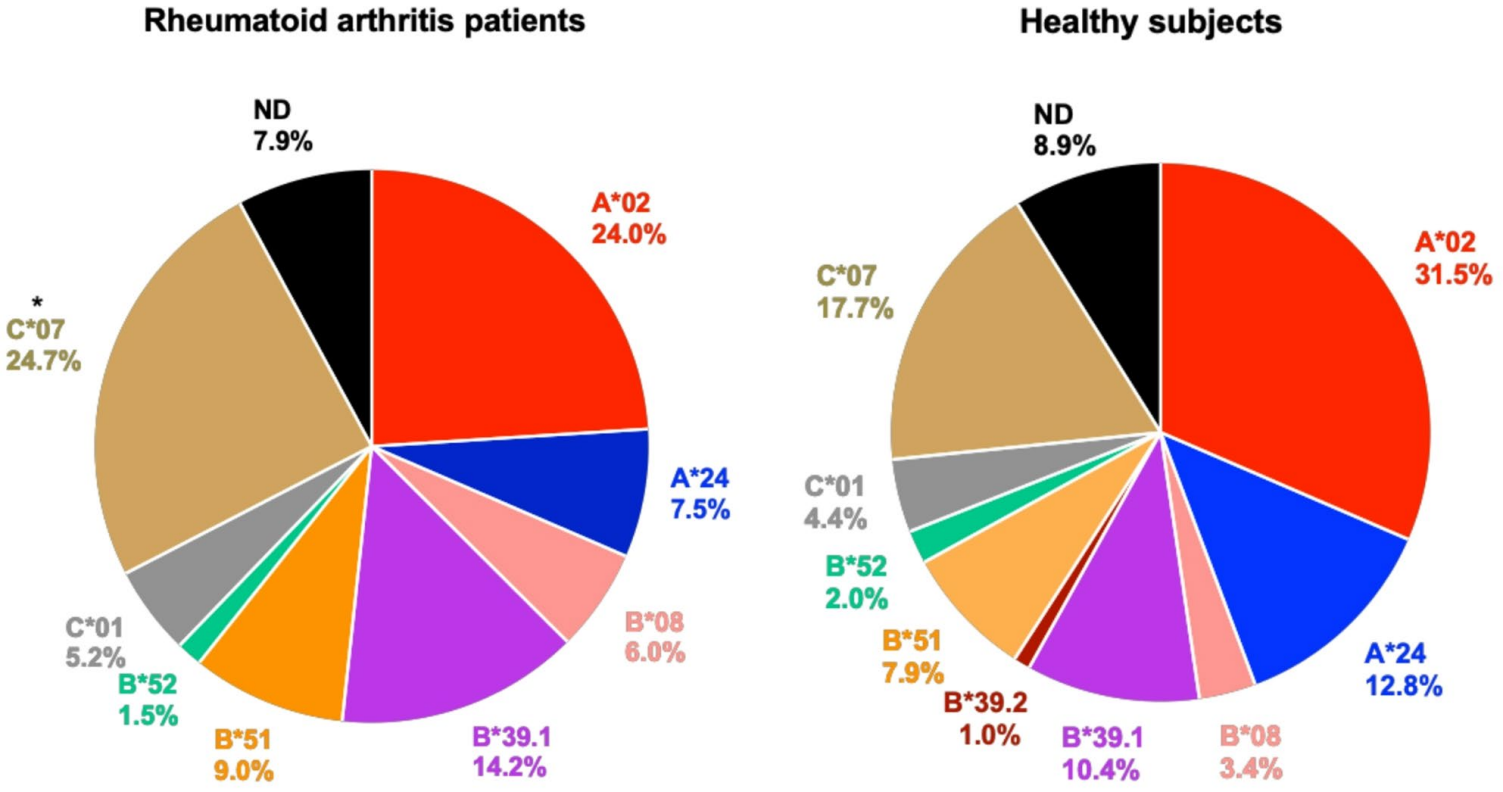
Distribution of *HLA-ABC* allele groups in Chilean rheumatoid arthritis (RA) patients and healthy subjects (HS). The *HLA-ABC* allele groups of interest were determined in a Chilean population of the Metropolitan region by polymerase chain reaction (PCR) using specific sequence primers targeting the HLA class I region (PCR-SSP). The cohorts were 135 RA patients and 122 HS. Differences between groups were assessed using Chi-square or Fisher’s exact tests. Statistically significant associations were subsequently evaluated using the Bonferroni correction; a significant difference was found only for the HLA-C*07 allele group (*p* = 0.015). Allele-group labels (e.g., HLA-A*02, -A*24, -B*08, -B*39.1, -B*39.2, -B*51, -B*52, -C*01, -C*07) correspond to low-resolution PCR-SSP groupings and do not represent specific International ImMunoGeneTics Information System (IMGT)/HLA alleles. In particular, B*39.1 and B*39.2 denote SSP-defined allele groups that include multiple HLA-B*39 and HLA-B*67 alleles, as detailed in Supplementary Table 1. ND indicates individuals for whom no allele group could be assigned by PCR-SSP using the primer sets applied in this study

DNA samples from RA patients (n = 135) and HS (n = 122) were typed for *HLA-ABC* allele groups by PCR-SSP. In RA patients, the highest allele group frequency corresponded to HLA-C*07 (24.7%), followed by HLA-A*02 (24.0%) and HLA-B*39.1 (14.2%). In contrast, in HS, the HLA-A*02 allele group presented the highest frequency (31.5%), followed by HLA-C*07 (17.7%) and HLA-A*24 (12.8%). When comparing the *HLA-ABC* allele groups frequencies between RA patients and HS, a statistically significant difference was observed for HLA-C*07 (OR = 2.29, *p* = 0.0015) and HLA-B*39.1 (OR = 1.88, *p* = 0.037) groups, in both cases being higher in RA patients (Table 1, Fig. 1). To eliminate the possibility of a Type I error, the Bonferroni correction was applied, resulting in a significant difference only for the HLA-C*07 allele group (*p* = 0.015).

**Table 1:** *HLA-ABC* allele group frequencies in Chilean rheumatoid arthritis (RA) patients and healthy subjects (HS)

*HLA-ABC* allele groups	RA patients		HS		*p-*value	Odds ratio (95% CI)
	Frequency		Frequency			
	%	n	%	n		
A*02	24.0	64	31.5	64	0.417	0.82 (0.5–1.3)
A*24	7.5	20	12.8	26	0.175	0.64 (0.4–1.2)
B*08	6.0	16	3.4	7	0.086	2.21 (0.9–5.7)
B*39.1	14.2	38	10.4	21	0.037*	1.88 (1.1–3.5)
B*39.2	0.0	0	1.0	2	0.224	0.00 (0.0–1.9)
B*51	9.0	24	7.9	16	0.303	1.43 (0.7–2.8)
B*52	1.5	4	2.0	4	1	0.9 (0.3–3.2)
C*01	5.2	14	4.4	9	0.401	1.45 (0.6–3.5)
C*07	24.7	66	17.7	36	0.0015**	2.29 (1.4–3.8)
N.D	7.9	21	8.9	18	0.657	1.17 (0.6–2.3)
Total^&^	100.0	267	100.0	203		

N.D., Represents the percentage of RA patients and HS who carried *HLA-ABC* allele groups not detected by the methodology used; CI, Confidence Interval

^&^The total numbers in the Table are higher than those of RA patients and HS since more than one *HLA-ABC* allele group is present in the same individual

*p-value ≤ 0.05 and **p-value ≤ 0.01, calculated by the Chi-square test or Fisher’s exact tests

Although the methodology used to classify the *HLA-ABC* allelic groups allowed us to partially determine the haplotypes carried by each participant in the study, without being able to define the specific alleles, we performed a genotypic frequency calculation using the available information from allelic group combinations for each of the three HLA-ABC *loci*. Thus, the most frequently found haplotypes in RA patients were HLA-A*02 ~ HLA-B*39.1 ~ HLA-C*07 (16.3%), HLA-A*24 ~ HLA-B*39.1 ~ HLA-C*07 (5.9%), and HLA-A*02 ~ HLA-B*51 ~ HLA-C*07 (5.9%), whereas in HS these haplotypes appeared at lower frequencies (7.4%, 2.5%, and 1.6%, respectively). The difference between RA patients and HS was significant for only the HLA-A*02 ~ HLA-B*39.1 ~ HLA-C*07 haplotype (*p* = 0.028) (Supplementary Table 2). However, after applying the Bonferroni correction, this difference lost its significance (*p* = 0.170).

### Distribution of *HLA-ABC* allele groups in RA patients carrying *HLA-DRB1* SE alleles

A subset of 60 RA patients carried at least one *HLA-DRB1* SE allele. Within this group, HLA-DRB1*14:02 was the most frequent allele (26.1%), followed by HLA-DRB1*04:05, HLA-DRB1*01:01 (18.8% each), HLA-DRB1*04:04 (17.4%), HLA-DRB1*04:01 (13.0%), and HLA-DRB1*10:01 (5.8%). In this subset of patients, we examined the distribution of *HLA-ABC* allele groups (Table 2). Depending on the specific *HLA-DRB1* SE allele, the most frequently observed *HLA-ABC* allele groups were HLA-C*07 (22.7–60.0%), HLA-A*02 (0–33.3%) and HLA-B*39.1 (0–16.7%), whereas HLA-B*39.2 and HLA-B*52 were the least frequent. The HLA-C*07 allele group presented the highest frequency in patients carrying HLA-DRB1*10:01 (60.0%), HLA-DRB1*04:05 (28.6%), HLA-DRB1*04:01 and HLA-DRB1*04:04 (27.8% each), HLA-DRB1*14:02 (25.7%), and HLA-DRB1*01:01 (22.2%) alleles. By contrast, HLA-A*02 showed its highest frequency among patients with HLA-DRB1*04:05 (33.3%), HLA-DRB1*14:02 (31.4%), HLA-DRB1*01:01 and HLA-DRB1*04:01 (22.2% each), and HLA-DRB1*04:04 (16.7%) alleles (Table 2).

**Table 2:** *HLA-ABC* allele groups in Chilean rheumatoid arthritis (RA) patients expressing *HLA-DRB1* Shared Epitope (SE) alleles

*HLA-DRB1* alleles	*HLA-ABC* allele groups	Frequency		*HLA-DRB1* alleles	*HLA-ABC* allele groups	Frequency	
		%	n			%	n
*01:01* (n = 13)	A*02	22.2	4	*04:01* (n = 9)	A*02	22.2	4
	A*24	0	0		A*24	16.7	3
	B*08	16.7	3		B*08	0	0
	B*39.1	11.1	2		B*39.1	16.7	3
	B*39.2	0	0		B*39.2	0	0
	B*51	0	0		B*51	5.6	1
	B*52	0	0		B*52	0	0
	C*01	11.1	2		C*01	5.6	1
	C*07	22.2	4		C*07	27.8	5
	N.D	16.7	3		N.D	5.6	1
	Total	100.0	18		Total	100	18
*04:04* (n = 12)	A*02	16.7	3	*04:05* (n = 13)	A*02	33.3	7
	A*24	0	0		A*24	4.8	1
	B*08	5.6	1		B*08	4.8	1
	B*39.1	16.7	3		B*39.1	9.5	2
	B*39.2	0	0		B*39.2	0	0
	B*51	0	0		B*51	4.8	1
	B*52	0	0		B*52	0	0
	C*01	5.6	1		C*01	9.5	2
	C*07	27.8	5		C*07	28.6	6
	N.D	27.8	5		N.D	4.8	1
	Total	100.0	18		Total	100	21
*10:01* (n = 4)	A*02	0	0	*14:02* (n = 18)	A*02	31.4	11
	A*24	0	0		A*24	8.6	3
	B*08	20.0	1		B*08	0	0
	B*39.1	0	0		B*39.1	14.3	5
	B*39.2	0	0		B*39.2	0	0
	B*51	0	0		B*51	8.6	3
	B*52	0	0		B*52	0	0
	C*01	0	0		C*01	2.9	1
	C*07	60.0	3		C*07	25.7	9
	N.D	20.0	1		N.D	8.6	3
	Total	100	5		Total	100	35

N.D., Represents the percentage of RA patients who carried *HLA-ABC* allele groups not detected by the methodology used

Among the haplotypes present in this RA sub-cohort, the haplotype HLA-A*02 ~ HLA-B*39.1 ~ HLA-C*07 ~ HLA-DRB1*14:02 was shown to be the most frequently distributed, representing 10.0%, followed by the haplotypes HLA-A*02 ~ HLA-B*39.1 ~ HLA-C*07 ~ HLA-DRB1*04:01 and HLA-A*24 ~ HLA-B*39.1 ~ HLA-C*07 ~ HLA-DRB1*14:02 with 3.3% each (result not shown).

## Discussion

The Chilean population arose primarily from the admixture of local Amerindian groups residing in South America with the Spanish colonizers beginning in the XVI century. Although Africans were brought as slaves early during the colonization, they contributed minimally to the genetic heritage of the current Chilean population [36]. Likewise, the native populations already inhabiting America before contact with Europeans were quite heterogeneous, comprising several Amerindian groups [37]. More recently, two studies of continental ancestry, one using 30 ancestry-informative SNPs [38] and another employing GeneChip Arrays [39], have reported that the current Chilean population has about 43% Amerindian, 55% European, and 2% African ancestry on average [38].

Regarding the distribution of HLA alleles or allele groups in the Chilean population, scarce studies have addressed this issue [32–34]. Among the available data, only a limited number of studies have focused on the distribution of HLA alleles or other SNPs in RA patients. These studies include investigations describing a weak association of a SNP at position − 308 of the tumor necrosis factor promoter and RA [40, 41], and reports linking the presence of the SE alleles HLA-DRB1*01:01, HLA-DRB1*04:01, HLA-DRB1*04:04, HLA-DRB1*10:01 [22, 23] and HLA-DRB1*04:08 [23], in addition to the non-SE allele group *HLA-DR9,* to increased disease RA risk [23, 42].

Although our cohorts of RA patients and HS are relatively small, the findings on the distribution of different *HLA-ABC* allele groups align well with previous reports for healthy individuals from diverse regions of the world, including Germany [26], the Netherlands [27], Tunisia [28], China [29], Colombia [30], Brazil [31] and Chile [33, 43]. Across these populations, HLA-A*02, HLA-B*39, and HLA-C*07 allele groups commonly predominate [26–31, 44]. Interestingly, we observed a higher frequency of the HLA-C*07 and HLA-B*39.1 allele groups in RA patients compared to HS, with OR values of 2.29 (*p* = 0.0015) and 1.88 (*p* = 0.037), respectively. However, when applying the Bonferroni correction for multiple comparisons, the significance was only maintained for the HLA-C*07 allele group (*p* = 0.015). The presence of this allele group, along with other genes, suggests that it could be a potential risk factor for the development of RA. However, the few studies using imputation for determining the haplotypes on databases of patients with RA of Caucasian or Asian origin have converged on the finding that there is only an association with RA for HLA-A, specifically the asparagine residue at position 77 (HLA-A Asn77), and for HLA-B, the aspartic acid residue at position 9 (HLA-B*8-Asp9). No associations have been reported so far with any allele of the HLA-C *locus* [17, 25, 45].

In addition, the frequencies of the *HLA-ABC* allele groups in HS described here were compared with those reported by two previous studies in very similar Chilean populations: the first by Castro-Santos et al., in a sample from the city of Talca, in the central region of Chile [33], and the second by Solloch et al., in a cohort of non-indigenous Chileans and a subgroup of subjects with Mapuche ancestry, half of whom were from the Santiago Metropolitan Region, and the rest from across the country [34]. For the most prevalent *HLA-ABC* allele groups in our study versus Castro-Santos et al. and Solloch et al., respectively, the allele frequencies were comparatively as follows: HLA-A*02 with 31.5% / 24.06% / 22.54%; HLA-A*24 with 12.8% / 10.00% / 10.22%; HLA-B*39.1 with 10.4% / 9.38% / 9.61%; HLA-B*51 with 7.9% / 6.88% / 7.13%; and HLA-C*07 with 17.7% / 22.19% / 18.71%. It can be observed that all the frequency percentages are very similar except for the HLA-A*02 allele group, which was considerably higher in our study. This difference could be explained since, in our case, the primers designed for this allele group amplify a larger number of specific alleles (Supplementary Table 1). Of particular importance is the consistency of HLA-C*07 frequencies across all datasets, which this concordance reinforces, highlighting the representativeness of our HS cohort. However, because those external datasets do not include RA patients, they cannot be used to support an association between HLA-C*07 and RA. Therefore, the potential association observed in our study should be interpreted with caution, particularly given the relatively small sample size, which remains a limitation of this work.

Our analysis of *HLA-ABC* haplotypes in a sub-group of patients with RA and HS, for whom we had at least one allelic group for the three HLA-ABC *loci*, indicated that of the six most representative haplotypes (Supplementary Table 2), the most prevalent in both RA patients and HS was the HLA-A*02 ~ HLA-B*39.1 ~ HLA-C*07 haplotype. This difference was significant for the RA group compared to the HS group; however, it disappeared after applying the Bonferroni correction for multiple comparisons. Our results are partially in agreement with those reported by Schafer et al., who described the haplotype HLA-A*02 ~ *HLA-B39* as the third most prevalent in a population of HS from the same Region of Chile [32]. Meanwhile, the agreement is greater with that published by Solloch et al., where the most predominant haplotype in a Chilean population of HS is HLA-A*02:01 ~ HLA-B*39:09 ~ HLA-C*07:02, taking into account that in this study the haplotyping was carried out in an allelic-specific way, so the haplotype found in our study, should include the alleles HLA-A*02:01, HLA-B*39:09 and HLA-C*07:02, among others of the respective allelic groups [34] (Supplementary Table 1). A similar situation occurs on the report by Castro-Santos et al., who found that the haplotype HLA-B*39:09 ~ HLA-C*07:02 is the most frequent in a population of healthy subjects from Talca, Chile [33].

There exists more clear evidence regarding the risk conferred by *HLA-ABC* alleles to other autoimmune diseases, with associations reported for HLA-B*27 and ankylosing spondylitis [46], HLA-C*06:02 and psoriasis [47], HLA-B*51 and Behçet’s disease [48], and HLA-A*29 with birdshot chorioretinopathy [49]. Even though a few studies have attempted to evaluate the participation of *HLA-ABC* alleles as a risk factor for RA, most of them have performed fine mapping of the HLA region in RA patients versus controls, ratifying the established role of the *HLA-DRB1* alleles and identifying additional *HLA-A* and *HLA-B* alleles associations, as well as particular amino acid residues involved as independent contributors. Thus, the HLA-A Asn77 and the HLA-B*8-Asp9 variants have been associated with a higher risk of developing RA in ACPA-positive Caucasian and East Asian subjects [17, 25, 45]. Likewise, in patients with ACPA-negative RA, it has been described that in the Chinese population, the *HLA-B27:04* allele is associated with the disease, while in the Indian population, the same occurs with the HLA-B molecule and a Valine at position 12 [17]. Our dataset´s sample size was insufficient to detect significant differences in the HLA-B*08 or HLA-A*02 allele groups (Table 1).

For a subset of RA patients included in this study, we had information about *HLA-DRB1* SE alleles typing, according to which, most patients carry at least one copy of an SE allele. The most frequent alleles among them were HLA-DRB1*04:01, HLA-DRB1*04:04 and HLA-DRB1*14:02, at frequencies of 16.1%, 13.9%, and 11.7%, respectively [50]. However, in the present study, the low number of individuals prevented the detection of any significant correlation between the *HLA-ABC* allele groups and specific *HLA-DRB1* SE alleles. It was only possible to note that the most common *HLA-ABC* groups (HLA-C*07, HLA-A*02 and HLA-B*39.1) appeared repeatedly in patients with different *HLA-DRB1* SE alleles (Table 2). By extending the haplotype analysis to include *HLA-DRB1* SE alleles from the RA sub-cohort, we found that the highest frequency was for the HLA-A*02 ~ HLA-B*39.1 ~ HLA-C*07 ~ HLA-DRB1*14:02 haplotype. Therefore, we are inclined to believe that the differences observed between the two study groups are more likely due to linkage disequilibrium of some *HLA-ABC* alleles, in this case HLA-C*07, with specific *HLA-DRB1* SE alleles, particularly with the HLA-DRB1*14:02, one of the most prevalent in the Chilean population, as we have described very recently [50], rather than a direct effect of the HLA-C*07 itself. Our haplotype analysis data are consistent with previous reports, showing that the most frequently represented HLA alleles are frequently found when studying extended haplotypes of classical HLA *loci* [44]. In order to understand the real impact of the HLA-C*07 allele group on RA susceptibility, it would be relevant in future studies to include a sub-cohort of RA patients who are *HLA-DRB1* SE negative, in order to define the presence of the HLA-C*07 allele group and its haplotype association with *HLA-DRB1* alleles, ideally in much larger cohort of patients.

It is important to mention that although RA prevalence varies only slightly across distinct geographic regions, the frequency of *HLA-DRB1* SE alleles differ significantly among ethnic groups [51]. Furthermore, most of the available information comes from studies conducted mainly in Caucasian and East Asian populations, with limited representation of the Latin American population. Furthermore, although the potential risk conferred by HLA alleles also depends on the presence or absence of ACPAs in RA patients, recent evidence indicates that the non-SE allele groups HLA-DRB1*09 and HLA-DRB1*15 can significantly influence ACPA levels, independently of ethnicity [52]. In our study, 91.8% of the RA patients were ACPA-positive, but the small number of subjects does not allow us to perform stratified analyses based on the ACPA status.

The knowledge of the risk conferred by *HLA-DRB1* alleles in RA has been crucial, first, to understand the pathogenesis of the disease, and second, to learn about the relationship between HLA molecules and autoantigenic peptides and the interaction of the peptide-HLA complexes with the T-cell antigen receptor of the autoreactive lymphocytes. Although RA is typically considered a CD4+ T cell-driven disease, which recognize autoantigenic peptides presented through HLA-DR molecules, there is growing evidence suggesting that CD8+ T cells may be involved in the immunopathogenesis of the disease [53–56]. While the role of CD8+ T cells in causing joint damage in RA remains unconfirmed, some autoantigenic peptides may bind more strongly to certain HLA-ABC molecules due to changes in key amino acid residues that make up the peptide-binding cleft, as may occur in HLA-B*08-Asp9 [17, 25, 45].

Therefore, investigating the involvement of *HLA-ABC* alleles in RA susceptibility is highly relevant. In our view, characterizing the distribution of these alleles in RA is pivotal for advancing the immunopeptidomic research, especially when prioritizing candidate peptides by theoretical affinity for particular *HLA-ABC* alleles. This approach becomes even more valuable given the scarcity of studies describing a possible association between RA and *HLA-ABC* alleles, as well as the importance of population-specific allele frequencies in designing effective immunological and translational investigations.

## Conclusions

In a Chilean cohort of RA patients from the Santiago Metropolitan Region, a significant increase in the frequency of the HLA-C*07 allele group was observed compared to HS, along with an increased frequency of the HLA-A*02 ~ HLA-B*39.1 ~ HLA-C*07 haplotype. Within a subset of RA patients, depending on the *HLA-DRB1* SE allele, the most frequently represented *HLA-ABC* allele groups were HLA-C*07, HLA-A*02, and HLA-B*39.1, which were found to be most predominantly associated with the HLA-DRB1*14:02 allele. Except for the HLA-C*07 and HLA-B*39.1 allele groups, the other *HLA-ABC* allele groups studied showed similar distributions in RA and HS patients, which is consistent with previous data from Chilean population and other populations of different ethnicities. Despite the importance of our results, we must acknowledge that this study has significant limitations, including the small size of the analyzed cohorts, which is inherent to the scope of this type of study. Furthermore, the methodology used here for HLA typing is of low resolution, which does not permit the identification of specific alleles and should be replaced by next-generation sequencing in future studies. Finally, these findings have provided relevant information for bioinformatic analyses of the HLA class I immunopeptidome and the potential presentation of autoantigens via HLA ABC molecules to autoreactive CD8+ T cells in RA.

## Supplementary Information

Below is the link to the electronic supplementary material.

Supplementary Material 1

## Data Availability

The datasets used and/or analyzed during the current study are available from the corresponding author upon reasonable request.

## Abbreviations

ACR: American College of Rheumatology
ACPAs: Anti-citrullinated protein antibodies
EDTA: Ethylenediaminetetraacetic acid
EULAR: European League Against Rheumatism
HS: Healthy subjects
HLA-ABC: HLA class I molecules
HLA: Human leukocyte antigen
OR: Odds ratio
PCR: Polymerase chain reaction
PCR-SSP: Polymerase chain reaction with sequence-specific primers
RA: Rheumatoid arthritis
HLADRB1 SE: “Shared Epitope” of the beta chain of class II HLA molecules
SNP: Single nucleotide polymorphism
