# Supplementary material for: Distribution of HLA-ABC allele groups in a cohort of Chilean rheumatoid arthritis patients and healthy individuals
Source: Biol Res. 2025 Dec 24;59:3. doi: 10.1186/s40659-025-00663-w (PMC12814593; doi:10.1186/s40659-025-00663-w)
Supplement: Supplementary file 1 — Supplementary Material 1 [file 40659_2025_663_MOESM1_ESM.docx]

**Supplementary Table 1.** *HLA-ABC* alleles and allele group detected by polymerase chain reaction (PCR) using specific primers.

| ***Loci*** | **Sense primer** | **Anti-sense primer** | **Allele groups** | ***HLA-ABC* alleles to be amplified by PCR** |
| --- | --- | --- | --- | --- |
|  | 5-TgCCAAgTggAgCACCCAA | 5-gCATCTTgCTCTgTgCAgAT |  | Internal control: third intron of the *HLA-DRB1* gene |
| **A** | 5-gTggATAgAgCAggAgggT | 5-CCAAgAgCgCAggTCCTCT | *A*02* | **02:01; *02:02; *02:03; *02:04; *02:05; *02:06; *02:07; *02:08; *02:09; *02:10; *02:11; *02:12; *02:13; *02:14; *02:15; *02:16; *02:17; *02:18; *02:19; *02:20; *02:21; *02:22; *02:23; *02:24; *02:25; *02:26.* |
|  | 5-ggCCggAgTATTgggACgA | 5-CCTCCAggTAggCTCTCTg | *A*24* | **24:02; *24:02102L; *24:03; *24:04; *24:05; *24:06; *24:07; *24:08; *24:09N; *24:10; *24:11N; *24:13; *24:14.* |
| **B** | 5-gACCggAACACACAgATCTT | 5-CCgCgCgCTCCAgCgTg | *B*08* | **08:01; *08:02; *08:03.* |
|  | 5-CCgAgAgAgCCTgCggAA | 5-CgTgCCCTCCAggTAggT | *B*39.1* | **39:011; *39:013; *39:021; *39:022; *39:03; *39:04; *39:05; *39:061; *39:062; *39:07; *39:08; *39:09; *39:10; *39:11; *39:12; *67:011; *67:012.* |
|  | 5-gACCggAACACACAgATCTA | 5-CgTgCCCTCCAggTAggT | *B*39.2* | **39:10; *67:011; *67:012.* |
|  | 5-ACCgAgAgAACCTgCggAT | 5-CgTTCAgggCgATgTAATCT | *B*51* | **51:011; *51:012; *51:021; *51:022; *51:03; *51:04; *51:05; *51:06; *51:07; *51:08; *51:09; *51:11N; *52:011; *52:012.* |
|  | 5-ACCgggAgACACAgATCTC | 5-CgTTCAgggCgATgTAATCT | *B*52* | **52:011; *52:012.* |
| **C** | 5-CACAgACTgACCgAgTgAg | 5-CCCCAggTCgCAgCCAC | *C*01* | **01:02; *01:03.* |
|  | 5-CCgCgggTATgACCAgTC | 5-CAgCCCCTCgTgCTgCAT | *C*07* | **07:01; *07:02; *07:03; *07:04; *07:05; *07:06; *07:07; *07:08.* |

**Supplementary Table 2.** *HLA-ABC* allele group haplotypes most prominently found in a cohort of Chilean rheumatoid arthritis (RA) patients and healthy subjects (HS).

| ***HLA-ABC* haplotypes** | **RA patients Frequency %** | **HS**  **Frequency %** | **p-value** | **Odds Ratio**  **95% CI** |
| --- | --- | --- | --- | --- |
| ***A*02~B*39.1~C*07*** | 16.3 | 7.3 | 0.028* | 1.42 (1.04 - 1.78) |
| ***A*24~B*39.1~C*07*** | 5.9 | 2.5 | 0.170 | 1.41 (0.83 - 1.83) |
| ***A*02~B*51~C*07*** | 5.9 | 1.6 | 0.076 | 1.56 (0.94 - 1.95) |
| ***A*02~B*08~C*07*** | 4.4 | 1.6 | 0.196 | 1.45 (0.78 -1.88) |
| ***A*02~B*51~C*01*** | 2.2 | 0.8 | 0.364 | 1.44 (0.57 - 1.92) |
| ***A*24~B*08~C*07*** | 0.8 | 0.8 | 0.999 | 0.95 (0.18 - 1.77) |

CI: Confident interval. *p-value ≤ 0.05 calculated by the Chi-square or Fisher`s exact tests; p-values adjusted using Bonferroni correction did not retain statistical significance.
